# Contemporary Mechanical Support Devices for Temporary and Long-Term Applications

**DOI:** 10.3390/bioengineering13020177

**Published:** 2026-02-03

**Authors:** Sriharsha Talapaneni, Sair Ahmad Tabraiz, Meghna Khandelwal, Shreya Avilala, Shanzil Shafqat, Sedem Dankwa, Chanseo Lee, Irbaz Hameed

**Affiliations:** Division of Cardiac Surgery, Department of Surgery, Yale University School of Medicine, New Haven, CT 06510, USA; sriharsha.talapaneni@yale.edu (S.T.);

**Keywords:** mechanical circulatory support, heart failure, cardiogenic shock, hemodynamic support

## Abstract

**Background**: Mechanical circulatory support (MCS) has revolutionized advanced heart failure and cardiogenic shock management, yet randomized controlled trials have failed to demonstrate consistent mortality benefits with temporary devices, and outcomes remain highly variable across institutions. **Methods**: This narrative review examines contemporary MCS devices, analyzing their hemodynamic principles, clinical outcomes, complications, and selection strategies. The published literature addressing MCS clinical applications and outcomes was reviewed, with reference lists examined to identify additional sources. **Results**: Temporary MCS devices demonstrate a persistent hemodynamic-survival paradox where improved hemodynamics fail to translate into mortality benefits in randomized trials. This disconnect reflects delayed intervention after irreversible organ damage, device complications offsetting hemodynamic gains, heterogeneous patient selection without phenotyping, timing challenges, and inadequate statistical power. Landmark trials provide definitive evidence against routine early VA-ECMO use, showing no survival advantage while significantly increasing complications. Optimal device selection requires integrating hemodynamic phenotyping with shock stage to match devices to pathophysiology, while biventricular failure presents the greatest challenge with substantially lower survival. For durable devices, third-generation systems demonstrate superior outcomes with dramatically reduced pump thrombosis and improved survival. Critically, multidisciplinary shock teams employing standardized protocols significantly reduce mortality beyond what devices alone achieve, with structured programs showing substantially improved survival compared to trials using similar devices without organized care systems. **Conclusions**: Mechanical circulatory support has transformed heart failure management, but optimal outcomes require integrating devices within structured care delivery systems. Success depends on comprehensive hemodynamic assessment, multidisciplinary team activation, protocolized device selection, standardized escalation and weaning strategies, and regionalized networks. The future lies in shifting focus from device innovation to implementation science, establishing quality metrics, developing precision medicine approaches, and conducting trials in phenotype-selected populations with protocolized care. This systems-of-care paradigm offers the most promising path toward translating technological advances into sustained mortality reduction.

## 1. Introduction

Heart failure is a heterogeneous clinical syndrome affecting approximately 26 million individuals worldwide, representing a significant public health challenge [1,2]. In the United States, it is a leading cause of hospitalization among those aged 65 and older, with healthcare costs reaching $31 billion in 2012 and projected to increase 127% by 2030 [3].

Cardiogenic shock, the most severe manifestation of acute heart failure, is characterized by cardiac pump dysfunction resulting in end-organ hypoperfusion and hypoxia [4]. Despite advances in cardiovascular care, mortality rates remain near 50%, particularly following acute myocardial infarction [5]. These persistently high mortality rates, combined with the growing population of patients with refractory heart failure unresponsive to guideline-directed medical therapy, have driven the development and refinement of mechanical circulatory support devices.

The modern era of mechanical circulatory support has been characterized by remarkable technological advances. Miniaturization of devices has improved portability and patient mobility, allowing individuals to maintain active lifestyles even while requiring mechanical support [6]. Improved biocompatibility through advanced materials has reduced complications such as thrombosis and hemolysis [7]. The transition from pulsatile to continuous-flow pumps, and more recently to magnetically levitated systems, has enhanced device durability and reduced mechanical complications [8]. Over 2000 mechanical circulatory support device implantations are performed annually in the United States, with utilization showing an upward trend [9].

However, a concerning paradox has emerged. Despite superior hemodynamic performance of contemporary temporary MCS devices compared to conventional therapies, randomized controlled trials have consistently failed to demonstrate mortality benefits. This persistent disconnect suggests a fundamental insight that device technology alone is insufficient. Instead, optimal results require integration of appropriate devices within structured care delivery systems. The National Cardiogenic Shock Initiative (NCSI), which implemented standardized protocols emphasizing multidisciplinary shock team activation, protocolized invasive hemodynamics, and early MCS deployment, reported 74% survival to hospital discharge, substantially exceeding the approximately 50% survival in contemporary randomized trials employing similar devices without structured care protocols [10,11]. The Critical Care Cardiology Trials Network study demonstrated that hospitals with dedicated shock teams had significantly reduced mortality (23% vs. 29%, adjusted OR 0.72, *p* = 0.016) compared to centers without shock teams [12].

This review provides a comprehensive analysis of mechanical circulatory support devices as components within integrated care systems. We examine how device capabilities interact with care delivery models including shock team structures, timing protocols, escalation strategies, and regionalized networks to influence patient outcomes. By analyzing evidence through this systems-of-care framework, we aim to explain persistent discordances between randomized trials and observational registries, provide actionable guidance for implementing structured shock programs, and identify implementation science priorities that may finally translate technological advances into sustained mortality reduction. The successful application of MCS requires understanding not only what devices can do, but how, when, and by whom they should be deployed within organized care systems.

## 2. Hemodynamic Monitoring and Patient Selection

The successful application of mechanical circulatory support requires careful patient selection guided by comprehensive hemodynamic assessment. Right heart catheterization provides critical objective data for risk stratification and device selection, measuring central venous pressure, pulmonary artery pressures, pulmonary capillary wedge pressure, and cardiac output [13]. Cardiac power output, calculated as the product of mean arterial pressure and cardiac output, has emerged as one of the strongest hemodynamic correlates of mortality in cardiogenic shock, with values below 0.6 watts predicting worse outcomes [14,15]. For right ventricular function assessment, the pulmonary artery pulsatility index (calculated as the difference between pulmonary artery systolic and diastolic pressures divided by central venous pressure) provides valuable insights, with values below 0.9 suggesting significant right ventricular impairment that may warrant dedicated right ventricular support [16,17].

The Society of Cardiovascular Angiography and Interventions classification stratifies patients into five stages (A through E) based on hemodynamics, biochemical markers, and clinical presentation, facilitating rapid identification of those who may benefit from mechanical circulatory support (Table 1) [18]. Cardiogenic shock can be further classified based on the affected ventricle: left ventricular dominant (elevated pulmonary capillary wedge pressure with normal central venous pressure), right ventricular dominant (elevated central venous pressure with normal pulmonary pressures), or biventricular failure (elevation of both) [19]. Notably, studies have shown that up to 40% of patients initially diagnosed with left ventricular-dominant shock actually have biventricular failure, highlighting the importance of comprehensive hemodynamic assessment [20]. Recent evidence from the Cardiogenic Shock Working Group demonstrates that patients who undergo complete hemodynamic profiling with right heart catheterization prior to mechanical circulatory support initiation experience lower in-hospital mortality compared to those without such comprehensive evaluation, underscoring the value of invasive hemodynamic monitoring in guiding therapeutic decisions [12,21].

## 3. Integrating Hemodynamic Assessment with Device Selection

**Figure 1:**
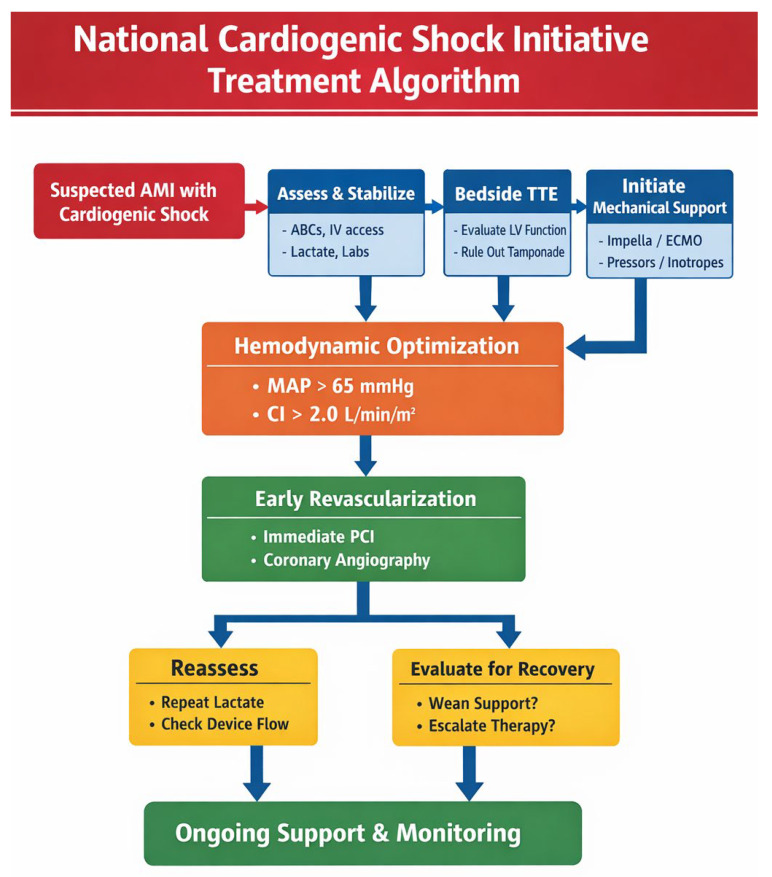
Cardiogenic Shock treatment initiative algorithm.

Mechanical circulatory support devices are designed to achieve three primary hemodynamic objectives that address the fundamental pathophysiology of cardiogenic shock and heart failure. First, they increase systemic perfusion, restoring adequate blood flow to vital organs and breaking the cycle of progressive organ dysfunction [22]. Second, they enhance coronary perfusion pressure, which is critical for myocardial recovery in cases of reversible cardiac injury [22]. Third, they reduce left ventricular filling pressures, wall stress, and myocardial oxygen consumption, thereby decreasing metabolic demands on the failing heart and creating conditions favorable for potential recovery [22]. The timing of mechanical circulatory support initiation has emerged as a critical determinant of outcome, with evidence suggesting that early deployment before irreversible end-organ damage improves survival. The National Cardiogenic Shock Initiative, which implemented a standardized treatment algorithm emphasizing rapid initiation of mechanical support prior to percutaneous coronary intervention in patients with acute myocardial infarction complicated by cardiogenic shock, reported survival to hospital discharge rates of 74%, substantially higher than historical controls (Figure 1) [10,11].

Furthermore, optimal device selection within shock team protocols requires integrating multiple hemodynamic parameters, shock phenotype, and clinical trajectory into a coherent decision-making framework:

SCAI Stage A (at-risk): Mechanical support is generally not indicated; focus remains on aggressive medical management and addressing the underlying etiology. Shock teams ensure close monitoring with defined triggers for escalation.

SCAI Stage B (beginning shock): Patients with hypotension but preserved cardiac index ≥ 2.2 L/min/m^2^ may respond to medical therapy alone. Shock team protocols mandate invasive hemodynamic monitoring within 1–2 h to guide escalation if deterioration occurs.

SCAI Stage C (classic shock): Device selection hinges on identifying the dominant hemodynamic derangement:

- LV-dominant shock (elevated PCWP > 18 mmHg, normal CVP < 12 mmHg, CPO ≤ 0.6 W): Devices providing active LV unloading are preferred. Impella CP/5.0 or TandemHeart offer superior LV decompression compared to IABP, though IABP may suffice in less severe presentations.
- RV-dominant shock (CVP > 12 mmHg, PAPi < 1.0, normal or low PCWP): Requires dedicated RV support with Impella RP or modified TandemHeart RV configuration.
- Biventricular failure (elevated CVP and PCWP, PAPi < 1.0, CPO ≤ 0.6 W): Presents the greatest challenge. Options include dual devices (Impella CP + RP), TandemHeart with subsequent RV support, or VA-ECMO, recognizing that ECMO alone does not directly unload the LV and may require adjunctive LV venting strategies.

SCAI Stage D (deteriorating) and E (extremis): VA-ECMO provides the most robust hemodynamic support and is often the only viable option for emergency stabilization. However, shock team protocols emphasize that even in these circumstances, the decision to deploy ECMO must weigh the very low likelihood of meaningful recovery against the high complication burden, particularly in patients with prolonged low-flow states exceeding 60 min, lactate > 5 mmol/L, or advanced age. Centers without ECMO expertise should activate transfer protocols to specialized shock centers rather than attempting inexperienced ECMO cannulation.

## 4. Types of Mechanical Circulatory Support Devices

Mechanical circulatory support devices can be classified based on several characteristics: duration of support (temporary versus durable), type of ventricular assistance provided (left ventricular, right ventricular, or biventricular), mechanism of action (volume displacement, axial flow, or centrifugal flow), and site of implantation (percutaneous versus surgical, intracorporeal versus extracorporeal). Understanding these classifications is essential for appropriate device selection based on individual patient characteristics and clinical scenarios.

## 5. Short-Term Mechanical Circulatory Support Devices

Short-term or temporary mechanical circulatory support devices are designed to provide hemodynamic assistance for periods ranging from hours to weeks [23,24]. These devices are typically used in acute settings such as cardiogenic shock, high-risk percutaneous coronary interventions, post-cardiotomy shock, or as bridges to more durable support or transplantation (Table 2) [23,24].

### 5.1. Intra-Aortic Balloon Pump

Intra-aortic balloon pump represents the oldest and most commonly used mechanical circulatory support device, introduced in the 1960s [27]. The device operates on counterpulsation principles, with a balloon in the descending thoracic aorta that inflates during diastole and deflates during systole [27]. Despite ease of bedside insertion via femoral access and widespread clinical familiarity, the intra-aortic balloon pump provides only modest hemodynamic support, typically increasing cardiac output by 0.5–1.0 L/min [27].

The landmark IABP-SHOCK II trial randomized 600 patients with acute myocardial infarction complicated by cardiogenic shock to receive either intra-aortic balloon pump or medical therapy alone, finding no difference in 30-day mortality (39.7% vs. 41.3%, *p* = 0.69), 12-month mortality (52% vs. 51%, *p* = 0.91), or 6-year mortality (66% vs. 67%, *p* = 0.80) [28,29]. A systematic review, encompassing 7 randomized trials with 790 patients, similarly demonstrated no mortality benefit with intra-aortic balloon pump compared to medical therapy alone (odds ratio 1.01, 95% CI 0.74–1.37, *p* = 0.96), though the device was associated with fewer vascular complications than more advanced systems (3.8% vs. 8.4%, *p* = 0.02) [30].

The BENCHMARK registry evaluated safety outcomes in 16,909 patients receiving intra-aortic balloon pump support, documenting major limb ischemia in 0.9%, major bleeding in 1.4%, and severe vascular injury requiring surgical repair in 0.3% of cases [31]. Complication rates were significantly higher in patients with diabetes (odds ratio 1.8, 95% CI 1.3–2.5, *p* < 0.001) and peripheral vascular disease (odds ratio 2.4, 95% CI 1.6–3.6, *p* < 0.001) [31]. These findings led contemporary European guidelines to recommend against routine intra-aortic balloon pump use in myocardial infarction complicated by cardiogenic shock [32]. Within shock team protocols, IABP is generally reserved for mechanical complications of MI (e.g., acute mitral regurgitation, ventricular septal defect) where augmentation of diastolic pressure is specifically beneficial, rather than as primary hemodynamic support.

### 5.2. Impella Devices

The Impella family comprises microaxial flow pumps that provide percutaneous mechanical circulatory support by actively translocating blood from the left ventricle to the ascending aorta [26]. These devices directly unload the left ventricle in a flow-dependent manner, reducing left ventricular end-diastolic pressure, pulmonary capillary wedge pressure, and right ventricular afterload while increasing mean arterial pressure, cardiac output, and cardiac power output [26].

Clinical evidence supporting Impella use comes primarily from observational studies and small randomized trials. The USpella Registry (n = 154) found that early Impella 2.5 initiation before percutaneous coronary intervention improved survival compared to post-procedural placement in acute myocardial infarction with cardiogenic shock (65% vs. 40%, *p* = 0.014) [33]. The ISAR-SHOCK trial (n = 26) demonstrated superior improvement in cardiac index with Impella 2.5 versus intra-aortic balloon pump (mean difference 0.49 L/min/m^2^, *p* = 0.02), but 30-day mortality was similar (46% vs. 50%, *p* = 0.84) [34]. The IMPRESS trial (n = 48) likewise found no mortality difference at 30 days (50% vs. 46%, *p* = 0.92) or 6 months (50% vs. 49%, *p* = 0.94) between Impella CP and intra-aortic balloon pump in cardiogenic shock [35].

For high-risk percutaneous coronary intervention, the PROTECT II trial (n = 452) was terminated early for futility, showing no difference in 30-day adverse events between Impella 2.5 and intra-aortic balloon pump (35.1% vs. 40.1%, *p* = 0.23) [36].

The cVAD registry (n = 3799) documented substantial complications: major bleeding in 26.8%, vascular complications requiring intervention in 7.4%, hemolysis necessitating device removal in 10.3%, and limb ischemia in 4.0% [37]. Larger bore devices (Impella 5.0/5.5) had significantly higher complication rates than smaller devices (2.5/CP): major bleeding 33.2% versus 22.4% (*p* < 0.001) and severe vascular complications 11.2% versus 5.1% (*p* < 0.001) [38].

### 5.3. TandemHeart

The TandemHeart is a percutaneous centrifugal continuous-flow pump that uses transseptal puncture to place a 21 Fr inflow cannula in the left atrium and a 15–17 Fr outflow cannula in the femoral artery, allowing parallel support with the native heart [39]. It is FDA-approved for 6 h but has CE marking for up to 30 days in Europe [40].

Two small randomized trials compared TandemHeart to intra-aortic balloon pump in cardiogenic shock. Burkhoff et al. (n = 33) found TandemHeart provided superior hemodynamic improvements: cardiac index increased by 0.5 L/min/m^2^ versus 0.1 L/min/m^2^ (*p* = 0.03), mean arterial pressure increased by 17 mmHg versus 5 mmHg (*p* = 0.02), and pulmonary capillary wedge pressure decreased by 8 mmHg versus 1 mmHg (*p* = 0.01) [39]. However, 30-day mortality was similar (43% vs. 47%, *p* = 0.86). Thiele et al. (n = 41) similarly demonstrated superior cardiac power output (0.68 W vs. 0.55 W, *p* < 0.05) and cardiac index (2.4 L/min/m^2^ vs. 1.9 L/min/m^2^, *p* < 0.05) with TandemHeart, but no mortality difference at 30 days (43% vs. 45%, *p* = 0.90) [41].

The multicenter registry by Kar et al. (n = 117) documented complications including severe bleeding in 19.3%, limb ischemia requiring intervention in 10.3%, transseptal puncture-related complications in 5.1%, and device malfunction in 2.6%. Major complications were associated with increased mortality (OR 3.2, 95% CI 1.4–7.3, *p* = 0.006), with complication rates rising significantly when support extended beyond 5 days (32% vs. 15%, *p* = 0.04) [42].

### 5.4. Extracorporeal Membrane Oxygenation

Extracorporeal membrane oxygenation provides both respiratory and hemodynamic support and has been used since the 1970s [43]. Two configurations exist, venovenous ECMO and venoarterial ECMO [44]. Venoarterial ECMO bypasses the cardiopulmonary circuit by withdrawing blood from the venous system, oxygenating it through a membrane oxygenator, and returning it to the arterial system [44,45].

The international multicenter study by Cheng et al., analyzing 4522 patients receiving venoarterial ECMO for cardiogenic shock, documented major bleeding in 40.8% of patients, lower limb ischemia in 16.9%, acute kidney injury requiring renal replacement therapy in 45.6%, neurological complications in 13.3%, and sepsis in 30.8% of cases [46]. The presence of any major complication was associated with significantly increased in-hospital mortality (OR 2.8, 95% CI 2.4–3.3, *p* < 0.001), and patients experiencing three or more complications had mortality rates exceeding 85% [46]. Additional complications include Harlequin syndrome (differential hypoxemia occurring in approximately 8.8% of cases), stroke (3.3–17.6% ischemic, 1.6–5% hemorrhagic), and limb ischemia requiring fasciotomy or amputation in 10–15% of peripheral cannulations [47].

Evidence supporting ECMO comes primarily from registries and observational studies. The Extracorporeal Life Support Organization registry reports survival-to-hospital-discharge rates of approximately 40% among adults receiving ECMO for cardiogenic shock and 29% for extracorporeal cardiopulmonary resuscitation [48]. The large French multicenter HELP-ECMO registry, which evaluated 414 patients with refractory cardiogenic shock receiving venoarterial ECMO, reported 30-day survival of 37.5% and 1-year survival of 32.1% [49]. Multivariate analysis identified several predictors of mortality, including lactate level greater than 5 mmol/L (hazard ratio 1.9, 95% CI 1.4–2.6, *p* < 0.001), duration of low-flow state exceeding 60 min (hazard ratio 1.6, 95% CI 1.2–2.2, *p* = 0.002), and age greater than 60 years (hazard ratio 1.4, 95% CI 1.1–1.9, *p* = 0.01) [49]. Notably, early initiation of ECMO support, defined as within 6 h of shock onset, was associated with improved survival compared to delayed initiation (42.3% vs. 28.7%, *p* = 0.003) [49].

The publication of two landmark randomized controlled trials has fundamentally challenged the expanding use of venoarterial ECMO in cardiogenic shock [50]. The ECLS-SHOCK trial, which enrolled 420 patients with acute myocardial infarction complicated by cardiogenic shock scheduled for early revascularization, demonstrated no survival benefit with early ECLS implementation [50]. At 30 days, mortality was 47.8% in the ECLS group versus 49.0% in the control group receiving usual medical treatment alone (relative risk 0.98, 95% CI 0.80–1.19, *p* = 0.81) [50]. Moreover, ECLS therapy was associated with significantly increased complications, including moderate or severe bleeding in 23.4% versus 9.6% of control patients (relative risk 2.44, 95% CI 1.50–3.95), and peripheral vascular complications requiring intervention occurred in 11.0% versus 3.8% (relative risk 2.86, 95% CI 1.31–6.25) [50]. Similarly, the ECMO-CS trial randomized 117 patients with rapidly deteriorating or severe cardiogenic shock to immediate VA-ECMO versus early conservative therapy, finding no significant difference in the composite primary endpoint of death, resuscitated cardiac arrest, or implementation of another mechanical circulatory support device at 30 days (63.8% versus 71.2%, hazard ratio 0.72, 95% CI 0.46–1.12, *p* = 0.21) [50]. Thirty-day all-cause mortality was also similar between groups at 50.0% versus 47.5% [50]. These neutral findings prompted lead investigator Holger Thiele to suggest that the steep increase in mechanical circulatory support use may not be justified, particularly given the high complication rates and resource-intensive nature of ECMO therapy. The trials’ results were particularly disappointing given the 77% prevalence of cardiac arrest prior to randomization in ECLS-SHOCK, suggesting patient selection and timing of intervention may be critical factors influencing outcomes.

### 5.5. Centrimage and Rotaflow Systems

The CentriMag left ventricular assist system and Rotaflow pump are magnetically levitated extracorporeal centrifugal flow pumps capable of providing flows up to 10 L/min [22]. The CentriMag is fully magnetically levitated without bearings or shafts, while the Rotaflow is suspended on sapphire bearings [22]. Both require surgical placement with cannulas positioned in the left atrium/aorta or right atrium/pulmonary artery depending on the ventricle being supported [22]. These pumps are FDA-approved for up to 6 h of left ventricular support and up to 30 days of right ventricular support, with CentriMag having CE marking for 30-day use for any indication [22]. The devices produce continuous non-pulsatile blood flow and are highly preload and afterload sensitive. An oxygenator can be spliced into the circuit to provide respiratory support when needed [22].

Evidence comes primarily from case series and registry data. Meta-analysis of studies using CentriMag showed 30-day survival rates ranging from 41% to 66%, with pooled survival of 52.4% (95% CI 46.8–58.0%, *p* < 0.001 for heterogeneity between studies), varying by indication [50]. The device has been commonly used for post-cardiotomy shock and as step-up therapy when hemodynamic support from peripheral devices proves inadequate.

The multicenter study by Takeda et al. evaluated 230 patients receiving CentriMag support for refractory cardiogenic shock and reported hospital discharge survival of 45.2% overall [51]. Outcomes varied significantly by treatment strategy, with patients bridged to durable ventricular assist devices or transplantation having substantially better outcomes compared to those intended for bridge to recovery (62.1% vs. 34.8%, *p* < 0.001) [51]. Duration of support ranged from 1 to 89 days with a median of 8 days, and survival rates declined significantly after 14 days of support (54.3% vs. 38.7%, *p* = 0.02), suggesting these devices are most effective for shorter-term support or as bridge to more definitive therapy [51].

Complications were substantial and included bleeding requiring reoperation in 29.6% of patients, stroke in 8.7%, infection in 24.3%, and acute kidney injury requiring dialysis in 38.3% of cases [52]. Importantly, patients who developed three or more major complications had substantially reduced survival compared to those with fewer complications (18.2% vs. 56.7%, *p* < 0.001), highlighting the cumulative impact of complications on outcomes [52]. The larger pump size and surgical implantation requirement make these systems less appealing than percutaneous alternatives when rapid deployment is needed, but they offer the advantage of very high flow rates and can support patients for extended periods when recovery or transplantation is anticipated.

### 5.6. Right Ventricular Support Devices

Right ventricular failure can complicate acute myocardial infarction, occur after left ventricular assist device implantation, or follow cardiac surgery. Dedicated right ventricular support devices have been developed to address this challenge.

The Impella RP is a 22 Fr catheter-based microaxial flow pump providing up to 4 L/min of flow [53]. Blood is aspirated from the inferior vena cava and ejected into the pulmonary artery, effectively unloading the right ventricle [53]. The device can be placed percutaneously and is approved for up to 14 days of support [53]. The RECOVER RIGHT trial evaluated the Impella RP in 30 patients with refractory right ventricular failure following cardiac surgery or left ventricular assist device implantation [54]. The device immediately reduced central venous pressure from a mean of 18.4 mmHg to 13.2 mmHg (*p* < 0.001) and improved cardiac index from 1.9 L/min/m^2^ to 2.5 L/min/m^2^ (*p* = 0.002), with an overall 30-day survival rate of 73.3% [54]. Survival was significantly higher in patients who achieved central venous pressure less than 15 mmHg within 24 h compared to those who did not (85.7% vs. 54.5%, *p* = 0.04). Complications are similar to other Impella devices, and the device is contraindicated in the presence of significant tricuspid or pulmonary regurgitation [54].

Alternative approaches to right ventricular support include modification of the TandemHeart system with right atrial inflow and pulmonary artery outflow (TandemHeart RVAD), and the TandemLife Protek Duo dual-lumen cannula with integrated right atrial and pulmonary artery access [55]. When respiratory failure coexists, these systems can incorporate an oxygenator, creating an oxy-RVAD configuration [55]. The THRIVE study retrospectively evaluated 46 patients receiving TandemHeart RVAD for acute right ventricular failure and found immediate improvements in hemodynamics, with mean pulmonary artery pressure decreasing from 38 mmHg to 28 mmHg (*p* < 0.001) and cardiac index increasing from 1.8 L/min/m^2^ to 2.3 L/min/m^2^ (*p* = 0.001), though in-hospital mortality remained elevated at 57% [35]. Patients bridged to durable therapy or recovery had significantly better outcomes than those receiving destination therapy (survival 62% vs. 28%, *p* = 0.02) [35].

Venoarterial ECMO can provide biventricular support and is sometimes used for right ventricular failure, though it does not directly unload the right ventricle and may worsen left ventricular distension if left ventricular function is preserved [56].

## 6. Understanding the Hemodynamic-Survival Paradox in Temporary MCS

The evidence base for temporary mechanical circulatory support reveals a persistent paradox, devices that demonstrably improve hemodynamic parameters fail to consistently improve survival in randomized controlled trials. Multiple trials have documented superior hemodynamic performance with advanced temporary MCS devices compared to conventional therapies, yet mortality remains unchanged. Impella devices achieve better cardiac index and cardiac power output than IABP but show similar 30-day mortality in ISAR-SHOCK, IMPRESS, and PROTECT II trials [34,35,36]. TandemHeart provides superior hemodynamic improvements versus IABP (cardiac index increased by 0.5 L/min/m^2^ vs. 0.1 L/min/m^2^, *p* = 0.03), yet 30-day mortality was identical (43% vs. 47%, *p* = 0.86) [39,41]. VA-ECMO provides the most robust cardiopulmonary support of any temporary device, yet demonstrates no survival benefit in adequately powered randomized trials.

The publication of two landmark randomized controlled trials has fundamentally challenged the expanding use of VA-ECMO in cardiogenic shock. The ECLS-SHOCK trial (n = 420) demonstrated no survival benefit with early ECLS implementation (mortality 47.8% vs. 49.0%, RR 0.98, 95% CI 0.80–1.19, *p* = 0.81), while ECLS therapy was associated with significantly increased complications: moderate or severe bleeding in 23.4% versus 9.6% (RR 2.44) and peripheral vascular complications requiring intervention in 11.0% versus 3.8% (RR 2.86) [50]. Similarly, the ECMO-CS trial (n = 117) found no significant difference in the composite primary endpoint at 30 days (63.8% versus 71.2%, HR 0.72, *p* = 0.21) [50]. These neutral findings establish clear practice-informing evidence: routine early VA-ECMO deployment provides no survival advantage while significantly increasing bleeding and vascular complications. Shock team protocols should therefore reserve ECMO for highly selected patients with refractory shock (SCAI Stage E or selected Stage D with refractory biventricular failure) rather than employing it as standard early intervention, especially in low-volume ECMO centers.

This disconnect reflects five fundamental challenges. First, delayed deployment after irreversible organ injury occurs when trials exceed 6–8 h from shock onset to device placement, allowing progressive cellular damage that hemodynamic support cannot reverse. Second, device complications offset hemodynamic benefits, as Impella complications (bleeding 27%, hemolysis 10%, vascular injury 7%) and ECMO complications (bleeding 41%, limb ischemia 17%, AKI 46%) may negate perfusion improvements [37,47]. Third, heterogeneous patient selection without phenotyping means Impella may benefit LV-dominant shock but harm biventricular failure by worsening RV afterload, yet trials made no such distinctions. Fourth, the timing paradox exists whereby intervening too early subjects recovering patients to complications while intervening too late misses the prevention window. Fifth, statistical power limitations with only approximately 200 patients total across Impella trials are adequate to detect only very large effects (>15% absolute reduction), potentially missing meaningful subgroup benefits.

The critical question centers on when, in whom, and within what care system devices should be deployed rather than whether they work at all. Devices may be effective when deployed early based on objective triggers (before irreversible damage), matched to shock phenotype through comprehensive hemodynamic assessment, integrated within multidisciplinary shock teams, supported by systematic complication prevention, and managed at high-volume centers. The NCSI’s 74% survival versus approximately 50% in trials using similar devices suggests care delivery systems matter as much as device selection [10,11].

Evidence-based recommendations with strong supporting evidence include reserving VA-ECMO for refractory shock only, performing comprehensive hemodynamic assessment (RHC with CPO and PAPi) before device selection, implementing systematic complication prevention, and using objective escalation and weaning triggers. Recommendations with moderate supporting evidence include deploying temporary MCS early in appropriate patients based on hemodynamic triggers, matching device to phenotype (LV-dominant with Impella or TandemHeart, RV-dominant with Impella RP, biventricular with dual devices or ECMO), and minimizing exposure through systematic weaning starting at 24 to 48 h. Areas of uncertainty include optimal hemodynamic thresholds for initiation, which specific patient subgroups benefit from which devices, and whether prophylactic MCS improves outcomes.

The future lies not in devices with greater hemodynamic capability, but in refining patient selection, optimizing timing, preventing complications, and integrating devices within evidence-based care systems. Only by addressing these challenges can superior hemodynamics translate into improved survival.

## 7. Long-Term or Durable Mechanical Circulatory Support Devices

Long-term or durable mechanical circulatory support devices represent a transformative advancement in the management of end-stage heart failure [57]. These devices are designed to provide sustained hemodynamic support for months to years, either as a bridge to cardiac transplantation or as permanent destination therapy for patients who are not transplant candidates [57]. The evolution of durable MCS technology has progressed through three generations, each offering improvements in durability, biocompatibility, and patient outcomes (Table 3) [58].

### 7.1. Patient Selection for Durable MCS

The decision to implant a durable mechanical circulatory support device requires careful patient selection guided by established criteria. The INTERMACS classification system stratifies patients with advanced heart failure into seven levels based on symptom severity and clinical trajectory [9,62]. Profiles 1 and 2 represent patients in critical cardiogenic shock or progressive decline despite inotropic support requiring intervention within hours to days. Profiles 3 and 4 include stable but inotrope-dependent patients or those with frequent symptoms at rest, with intervention recommended within weeks to months [9]. Profiles 5 through 7 encompass patients with varying degrees of exertional limitation who may not currently require mechanical support [9,57]. While durable devices were initially developed primarily for profiles 1 and 2, contemporary practice has expanded to include profiles 3 and 4, reflecting improved outcomes and growing confidence in device technology [57].

In the United States, Centers for Medicare and Medicaid Services criteria for LVAD coverage include left ventricular ejection fraction ≤ 25%, peak oxygen consumption ≤ 14 mL/kg/min, NYHA class IV symptoms, and cardiac index < 2.2 L/min/m^2^ despite optimization on guideline-directed medical therapy for 45 of the last 60 days [63]. Absolute contraindications include irreversible end-organ dysfunction, severe mental illness, medication non-adherence, and severe right ventricular dysfunction without support options. Relative contraindications include advanced age over 80 years, morbid obesity, active infection, untreated malignancy, and recent substance use [64].

The two primary indications for durable MCS implantation are bridge to transplantation for patients who are transplant candidates requiring mechanical support while awaiting a donor organ, and destination therapy for patients with end-stage heart failure who are not transplant candidates due to age, comorbidities, or other factors, for whom the device serves as permanent cardiac replacement therapy [65].

### 7.2. Evolution of Pump Technology

The technological evolution of durable ventricular assist devices has progressed through three distinct generations. First-generation devices were pulsatile volume displacement pumps including the HeartMate XVE, Thoratec Paracorporeal Ventricular Assist Device, and Novacor, which successfully demonstrated the feasibility of long-term mechanical support but were limited by large size, high rates of mechanical failure, and frequent maintenance needs [66]. Second-generation devices introduced continuous axial flow pumps with mechanical bearings, such as the HeartMate II, Jarvik 2000, and MicroMed DeBakey VAD, featuring rotary pumps with impellers rotating at 6000 to 15,000 revolutions per minute [22]. The transition to continuous-flow technology resulted in significant miniaturization, improved durability, and reduced surgical complexity [67]. Between 2006 and 2017, second-generation devices accounted for approximately 78% of all durable mechanical circulatory support device implantations according to INTERMACS registry data [9]. However, mechanical bearings created areas of blood stasis and turbulence, predisposing to thrombosis and hemolysis, which drove the development of third-generation technology [68].

Third-generation devices employ centrifugal pumps with magnetic or hydrodynamic levitation, eliminating mechanical bearings entirely [69]. Examples include the HeartMate 3 and HeartWare HVAD. The fully magnetically or hydrodynamically levitated impeller creates a contactless pumping mechanism that significantly reduces areas of blood stasis, thereby decreasing thrombosis and hemolysis [70]. These devices generate continuous flow through a centrifugal rotor with a single moving part suspended in an electromagnetic field, creating a Coanda effect wherein blood swirls against the pump housing with minimal shear stress [70]. Compared to axial flow pumps, centrifugal pumps are more sensitive to afterload, less prone to suction events, and generate greater pulsatility [71]. Currently, more than 90% of durable mechanical circulatory support devices implanted in the United States are continuous-flow devices, with the remaining 10% consisting of total artificial hearts, reflecting the near-complete transition away from pulsatile technology [58].

### 7.3. HeartMate II

The HeartMate II represents the most widely studied and implanted second-generation left ventricular assist device [67]. It is a continuous axial flow pump featuring a titanium-coated rotor capable of generating flows up to 10 L/min at speeds between 6000 and 10,000 revolutions per minute [67]. The device received FDA approval for bridge to transplantation in April 2008 and for destination therapy in January 2010 [67].

The pivotal HeartMate II trial compared this device to the first-generation HeartMate XVE in patients deemed ineligible for cardiac transplantation [67]. Two-year survival with HeartMate II was 58% compared to 24% with HeartMate XVE (*p* < 0.001), representing more than a doubling of survival [67]. This dramatic improvement established the superiority of continuous-flow technology as the new standard of care and justified widespread adoption of continuous-flow devices.

The ROADMAP study was a prospective non-randomized trial comparing HeartMate II with optimal medical therapy in patients with advanced heart failure who were less critically ill than typical device candidates [72]. Survival at one year was 85% with the device compared to 73% with medical therapy alone (*p* = 0.03), and at two years was 70% versus 58% (*p* = 0.06), respectively [72]. Quality of life measures showed substantial improvements with device therapy across multiple domains. These findings supported expansion of left ventricular assist device use beyond patients in cardiogenic shock to include more stable patients with advanced heart failure, broadening the eligible patient population significantly.

Despite excellent survival outcomes, pump thrombosis emerged as a significant concern with the HeartMate II over time. The PREVENT trial investigated strategies to minimize pump thrombosis and found that adherence to therapeutic anticoagulation recommendations was associated with significantly lower thrombosis risk [68]. At three months, the incidence of pump thrombosis was 2.9%, increasing to 4.8% at six months, demonstrating that thrombosis risk accumulates over time [68]. When pump thrombosis occurs, it often necessitates device exchange, a high-risk surgical procedure, or heart transplantation if the patient is a candidate. This complication ultimately drove the development of third-generation devices with improved hemocompatibility.

### 7.4. HeartMate 3

The HeartMate 3 represents a major advancement as a third-generation fully magnetically levitated centrifugal flow pump designed to address the limitations of earlier devices [69]. The device can generate flows up to 5 L/min and features several key innovations designed to reduce complications, particularly thrombosis [69].

The device received CE marking in Europe in 2015 and FDA approval in the United States in 2018 [73]. The HM3 CE mark trial was a prospective single-arm study demonstrating one-year mortality of 18%, with no cases of pump thrombosis or pump malfunction (0% pump thrombosis, *p* < 0.001 compared to historical HeartMate II data), and significant improvements in quality of life and functional capacity over time [69]. These results exceeded historical controls and suggested that the device design successfully addressed the thrombosis issues that plagued earlier generation pumps.

The landmark MOMENTUM 3 trial demonstrated clear superiority over HeartMate II, with two-year survival free of disabling stroke or device removal for malfunction/thrombosis of 76.9% versus 56.0% (*p* = 0.001) [74]. While overall survival rates were similar between groups (79.5% vs. 81.0%, *p* = 0.49), the HeartMate 3 had markedly fewer device-related complications [74]. Most notably, pump thrombosis occurred in only 1.4% with HeartMate 3 compared to 13.2% with HeartMate II (HR 0.10, 95% CI 0.03–0.31, *p* < 0.001), representing a 10-fold reduction [74]. Additionally, hospitalization rates for device-related complications were lower with HeartMate 3 (41.7% vs. 51.1%, *p* = 0.04), suggesting improved overall device reliability and safety [74].

Five-year data showed sustained benefits with improved survival (58.4% vs. 43.7%, *p* = 0.02) and superior freedom from pump thrombosis (98.2% vs. 84.8%, *p* < 0.001) [75]. The improved safety profile has enabled investigation of reduced anticoagulation strategies in the MAGNETUM trials [76]. HeartMate 3 is now the dominant durable LVAD platform worldwide, accounting for over 90% of contemporary implants [75]. Superior treatment effects were observed regardless of whether the indication was bridge to transplantation or destination therapy (*p* for interaction = 0.62), supporting the broad applicability of the device across patient populations [75].

The improved safety profile of HeartMate 3 has prompted investigation into reduced anticoagulation strategies. The MAGNETUM 1 pilot trial evaluated targeting INR 1.5 to 1.9, achieving the primary endpoint of survival free of pump thrombosis, disabling stroke, and major bleeding at six months in 93% of participants with no cases of pump thrombosis (0%, 95% CI 0–5.4%), though larger randomized trials are needed before this can be recommended as standard practice. A class I device recall occurred in May 2018 due to outflow graft twisting causing device malfunction and patient deaths, prompting manufacturer design modifications and surgical technique recommendations that have resolved this complication in subsequent devices [77].

### 7.5. HeartWare HVAD

The HeartWare Ventricular Assist Device (HVAD) was a third-generation continuous-flow centrifugal pump approved by the FDA for bridge to transplantation in November 2012 and for destination therapy in September 2017 [78]. The device could generate flows up to 10 L/min and featured a compact design that allowed intrapericardial implantation without the need for an abdominal pump pocket, potentially reducing infection risk and surgical complexity [78].

The ADVANCE trial evaluated HeartWare HVAD as a bridge to transplantation, comparing outcomes to contemporary controls from the INTERMACS registry who received other commercially available pumps. The primary outcome of survival to 180 days without device change or transplantation was similar between groups (90.7% for HVAD versus 90.1% for controls, *p* = 0.86), supporting the device’s approval for bridge to transplantation and suggesting non-inferior short-term outcomes [78].

The ENDURANCE trial compared HVAD to HeartMate II in 446 destination therapy patients, finding comparable overall survival (59.1% vs. 58.3%, *p* = 0.84) but significantly higher stroke rates with HVAD (29.7% vs. 12.1%, HR 2.71, *p* < 0.001), representing more than a doubling of stroke risk, alongside increased right heart failure requiring RVAD support (20.4% vs. 11.0%, *p* = 0.008) and sepsis (22.2% vs. 13.0%, *p* = 0.02) [79]. A subsequent registry analysis of 13,704 patients found HVAD was associated with higher two-year mortality compared to HeartMate 3 (43.8% vs. 35.2%, HR 1.28, *p* < 0.001) and increased stroke rates (18.4% vs. 10.2%, HR 1.85, *p* < 0.001). These accumulating safety concerns led to voluntary market withdrawal of the HVAD system by Medtronic in June 2021, citing observed survival benefit with alternative devices, particularly HeartMate 3, underscoring the importance of ongoing post-market surveillance and head-to-head comparative trials.

### 7.6. Total Artificial Heart

The SynCardia Total Artificial Heart completely replaces the native ventricles with two pneumatic artificial ventricles, each with 70 mL stroke volume, capable of generating flows up to 9.5 L/min with four mechanical valves ensuring unidirectional flow [80]. Approved only for bridge to transplantation in the United States, it requires body surface area > 1.7 m^2^ and thoracic diameter ≥ 10 cm [80]. A non-randomized study found one-year survival of 70% with Total Artificial Heart versus 31% in controls (*p* < 0.001), with post-transplant survival comparable to LVAD-bridged patients (86% vs. 89%, *p* = 0.58) [80].

A retrospective study of 47 patients supported for >1 year reported 72% successfully transplanted with median support duration of 554 days (range 365–1373 days) [81]. Major complications included systemic infections (53%), driveline infections (27%), thromboembolic events (19%), hemorrhagic events (14%), stroke rate of 0.083 per patient-year, and device failure (10%), with two deaths (4%) from membrane rupture [81]. Postoperative renal replacement therapy was required in 26% of patients [81]. Patients with BSA ≤ 1.8 m^2^ experienced significantly higher mortality (63% vs. 14%, *p* = 0.005), hemorrhagic events (45% vs. 5.5%, *p* = 0.009), and systemic infections (90% vs. 41%, *p* = 0.008) compared to those with BSA > 1.8 m^2^ [81].

The Total Artificial Heart is indicated for biventricular failure patients who are not candidates for LVAD alone or have contraindications to biventricular assist device support, or in cases of complex cardiac anatomy precluding VAD placement [82]. The newer Aeson by Carmat has received CE marking in Europe and incorporates bovine pericardial tissue, physiologic demand sensors, and pulsatile flow, with early feasibility study (n = 10) suggesting improved biocompatibility with lower C-reactive protein levels at one month (42 mg/L vs. 87 mg/L, *p* = 0.03), though long-term data are still being collected and FDA approval has not been obtained [83].

### 7.7. Biventricular Assist Devices

Biventricular support can be achieved through Total Artificial Heart implantation, placement of two left ventricular assist devices configured for both ventricles, or use of a left ventricular assist device with a temporary right ventricular assist device. The need arises in severe biventricular failure where isolated left ventricular support would be insufficient and would likely worsen right ventricular failure.

INTERMACS registry data show one-year survival with biventricular support is approximately 50% compared to 82% with isolated left ventricular assist devices (*p* < 0.001) [84]. A significant challenge is the lack of FDA-approved durable right ventricular assist devices, as the Impella RP is approved only for temporary support up to 14 days. Krabatsch et al. evaluating 99 patients with biventricular HeartMate II support reported one-year survival of 52%, with stroke in 24% and device-related infection in 38% [85]. Recent studies of dual HeartMate 3 implants showed survival rates at 18 months ranging from 54% to 91% depending on center [86]. A single-center series of 23 patients reported one-year survival of 65%, with right ventricular assist device weaning successful in 26% (*p* = 0.04 for weaning success prediction based on central venous pressure < 12 mmHg) [87].

The timing of right ventricular assist device placement is critical. A single-center study of 266 patients found planned biventricular placement was associated with improved survival to hospital discharge compared to delayed placement (51% vs. 29%, *p* = 0.0459). Reoperation rates (62% vs. 75%) and need for postoperative hemodialysis (31% vs. 39%) were similar between the biventricular groups [88]. Biventricular mechanical support remains challenging with significantly lower survival rates compared to isolated left ventricular support, and outcomes are improved when biventricular support is planned rather than delayed. Future studies should focus on developing FDA-approved durable right ventricular assist devices and refining patient selection criteria to identify which patients may successfully wean from right ventricular support.

## 8. Beyond Device Technology

While technological advances have improved device performance, the persistent gap between improved hemodynamics and survival benefit in randomized trials suggests that device technology alone is insufficient. The National Cardiogenic Shock Initiative (NCSI) implemented standardized protocols emphasizing rapid shock team activation, protocolized hemodynamic assessment within 1 to 2 h, and early MCS deployment before PCI, achieving 74% survival to hospital discharge compared to approximately 50% in contemporary randomized trials using similar devices without structured protocols [10,11]. The Critical Care Cardiology Trials Network study of 188 hospitals found centers with dedicated shock teams had significantly reduced mortality (23% vs. 29%, adjusted OR 0.72, *p* = 0.016), higher pulmonary artery catheter utilization (78% vs. 52%, *p* < 0.001), more frequent cardiac power output measurement (83% vs. 61%, *p* < 0.001), shorter time to intervention (median 3.2 vs. 5.8 h, *p* < 0.001), and lower inappropriate device selection rates (8% vs. 19%, *p* = 0.003) [12]. A European registry of 2465 patients showed 30-day mortality decreased from 48% to 39% (adjusted OR 0.68, *p* = 0.001) after implementing structured shock team protocols [12].

Successful shock team implementation requires rapid activation systems with target response time under 15 min, multidisciplinary teams including interventional cardiology, heart failure, cardiac surgery, critical care, and specialized nursing, protocolized right heart catheterization within 1 to 2 h measuring CVP, PA pressures, PCWP, cardiac output, CPO, and PAPi with serial reassessment every 4 to 6 h for stable patients or every 1 to 2 h for deteriorating patients, and standardized device selection algorithms matching phenotype to device (LV-dominant shock with CPO under 0.6 W receives Impella CP or TandemHeart, RV-dominant shock with PAPi below 0.9 receives Impella RP, biventricular failure receives dual devices or VA-ECMO with LV venting). The SCAI Working Group demonstrated 30% of patients presenting in Stage B/C progress to Stage D/E within 24 h, emphasizing need for continuous monitoring [18]. Shock team protocols use objective triggers (CPO under 0.6 W, lactate over 2 mmol/L with rising trend, PAPi below 0.9 for RV support) rather than subjective assessment. Systematic weaning protocols mandate assessment starting at 24 to 48 h with readiness criteria including MAP over 65 mmHg on minimal vasopressors, lactate below 2 mmol/L, improving organ function, and improved ventricular function on echocardiography, recognizing that support beyond 14 days requires definitive transition to durable support, transplant, or withdrawal.

Durable MCS success requires comprehensive longitudinal care beyond the operating room. Immediate post-operative management (days 0 to 7) focuses on hemodynamic optimization (target MAP 70 to 80 mmHg, ramp studies days 3 to 5), RV failure prevention (aggressive diuresis targeting CVP below 12 mmHg, pulmonary vasodilators, early temporary RVAD if PAPi below 0.9 and CVP over 15 mmHg), anticoagulation initiation (unfractionated heparin at 12 to 24 h transitioning to warfarin with target INR 2.0 to 3.0, aspirin 81 mg daily), and surveillance with serial echocardiography and device interrogation. Long-term management requires multidisciplinary clinics with LVAD coordinators, cardiologists, surgeons, anticoagulation specialists, social workers, and nutritionists, following routine schedules (weekly visits weeks 2 to 4, every 2 weeks months 2 to 3, monthly months 4 to 12, every 2 to 3 months after year 1) with quarterly echocardiography, strict INR monitoring (target 2.0 to 3.0), blood pressure control (target MAP 70 to 80 mmHg), driveline care education, and psychosocial support (depression present in 30% to 40% of recipients). Late complications requiring systematic surveillance include gastrointestinal bleeding (annual incidence 20% to 40%) from arteriovenous malformations managed with endoscopic ablation and octreotide, driveline infections (incidence 10% to 30%) requiring prolonged IV antibiotics and often chronic suppressive therapy, stroke (annual incidence 5% to 10%) requiring strict anticoagulation control and BP management, pump thrombosis (below 2% with HeartMate 3) presenting with rising power consumption and hemolysis managed with thrombolysis or urgent pump exchange with mortality 20% to 30%, and aortic insufficiency (progressive in 20% to 30%) monitored with serial echocardiography.

The 2022 AHA/ACC/HFSA guidelines recommend early triage of refractory shock to Level 1 centers, recognizing regionalized hub-and-spoke models are necessary [18]. Level 1 centers provide 24/7 advanced MCS availability, cardiac surgery with transplant expertise, dedicated shock teams, and over 50 MCS cases/year. Level 2 centers offer IABP and possibly Impella with ability to stabilize and transfer. Level 3 centers provide PCI capability, IABP placement, and rapid transfer initiation. Studies show patients at Level 2/3 centers managed via integrated protocols with Level 1 hubs have similar outcomes to direct Level 1 admissions (mortality 43% vs. 42%, *p* = 0.78, time to MCS 4.2 vs. 3.8 h, *p* = 0.31) [12]. Despite evidence, only 32% of U.S. hospitals with MCS capability have formal shock team protocols, representing a critical implementation gap driven by resource constraints, institutional silos, absence of standardized quality metrics, knowledge gaps, and resistance to protocolization [12].

## 9. Conclusions

Mechanical circulatory support has fundamentally transformed heart failure management, yet a critical paradox persists. Despite remarkable technological advances, randomized trials consistently fail to demonstrate mortality benefits with temporary MCS devices, revealing that device technology alone is insufficient. Emerging evidence demonstrates that optimal outcomes require integration of appropriate devices within structured care delivery systems. The National Cardiogenic Shock Initiative and Critical Care Cardiology Trials Network studies show that multidisciplinary shock teams employing standardized protocols significantly reduce mortality compared to ad hoc management, explaining the discordance between neutral randomized trials and improved observational outcomes.

For durable devices, third-generation systems demonstrate superior outcomes with substantially reduced pump thrombosis rates, yet long-term success requires comprehensive longitudinal care addressing anticoagulation, infection surveillance, and late complications. Critical challenges persist, including substantially lower survival with biventricular support compared to isolated LVAD, lack of FDA-approved durable right ventricular assist devices, and implementation gaps with only a minority of hospitals having formal shock team protocols despite compelling evidence.

The future of mechanical circulatory support lies not primarily in developing devices with greater hemodynamic capability, but in implementing structured care delivery systems that optimize deployment of existing technology. Success requires shifting focus from device innovation to implementation science, establishing standardized quality metrics, developing precision medicine approaches, addressing the durable RV support gap, and conducting adequately powered trials in phenotype-selected populations with protocolized care. Just as modern trauma care succeeded through implementing trauma systems rather than inventing new surgical techniques, modern shock care will advance through systematic implementation of evidence-based care delivery models. This systems-of-care paradigm represents the frontier of MCS optimization and offers the most promising path toward translating technological advances into sustained mortality reduction.

## Figures and Tables

**Table 1:** Society of Cardiovascular Angiography and Interventions Classification of Cardiogenic Shock.

Stage	Description	Key Hemodynamics	Biochemical Markers	Clinical Features	Device Selection
A: At Risk	Not currently in shock but at risk	Normotensive <br> CI ≥ 2.5 L/min/m^2^	Normal lactate and renal function	STEMI, NSTEMI, or acute HF at risk of progressing to shock	Monitoring
B:Beginning	Early shock	SBP < 90 mmHg or MAP < 60 mmHg <br> CI ≥ 2.2 L/min/m^2^	Normal lactate <br> Elevated BNP	Hypotension or tachycardia without hypoperfusion	Hours to days
C: Classic	Established shock	SBP < 90 mmHg <br> CI < 2.2 L/min/m^2^ <br> CPO ≤ 0.6 W <br> PAPi < 1.85	Lactate ≥ 2 mmol/L <br> Rising creatinine	Hypoperfusion requiring vasopressors or MCS	Within hours
D: Deteriorating	Worsening shock	Stage C criteria + deteriorating	Rising lactate and creatinine	Failing to respond to initial interventions	Immediate escalation
E: Extremis	Cardiac arrest	Circulatory collapse	pH ≤ 7.2 <br> Lactate ≥ 5 mmol/L	Cardiac arrest with CPR or requiring ECLS	Emergent

Abbreviations: BNP = B-type natriuretic peptide; CI = cardiac index; CPO = cardiac power output; CPR = cardiopulmonary resuscitation; ECLS = extracorporeal life support; HF = heart failure; MAP = mean arterial pressure; MCS = mechanical circulatory support; NSTEMI = non-ST elevation myocardial infarction; PAPi = pulmonary artery pulsatility index; SBP = systolic blood pressure; STEMI = ST elevation myocardial infarction.

**Table 2:** Short-Term Mechanical Circulatory Support Devices in Acute Cardiogenic Shock. “↑” is Increases, “↓” is decreases.

Device	Mechanism of Support	Typical Flow (L/min)	Primary Hemodynamic Effects	Key Indications	Major Complications	Role in NCSI Algorithm
Intra-Aortic Balloon Pump (IABP)	Diastolic counterpulsation	0.5–1.0	↑ Coronary perfusion, modest ↑ CO, ↓ afterload	AMI-CS (historical), mechanical complications of MI	Vascular injury, bleeding, limb ischemia (low) [25]	Limited role; not favored due to lack of mortality benefit
Impella 2.5/CP	Microaxial LV-to-aorta flow	2.5–4.0	LV unloading, ↓ PCWP, ↑ MAP, ↑ CPO	AMI-CS, high-risk PCI	Bleeding, hemolysis, vascular complications [26]	Preferred early support prior to PCI
Impella 5.0/5.5	Surgically placed microaxial LV support	5.0–5.5	Robust LV unloading, ↑ CO	Severe CS, bridge to decision/recovery	Higher bleeding & vascular risk [26]	Escalation strategy when percutaneous support insufficient
TandemHeart	LA-to-femoral artery centrifugal pump	4.0–5.0	↓ PCWP, ↑ CI, ↑ MAP	Refractory LV-dominant shock	Bleeding, limb ischemia, transseptal complications [26]	Alternative LV unloading strategy
VA-ECMO	Extracorporeal cardiopulmonary bypass	4.0–7.0	Full cardiopulmonary support, ↑ MAP	Profound shock, cardiac arrest	LV distension, bleeding, stroke, limb ischemia [25]	Rescue therapy; often combined with LV unloading
ECpella (VA-ECMO + Impella)	ECMO with active LV unloading	Variable	Maintains perfusion + prevents LV distension	Severe biventricular failure	High bleeding & complexity [25]	Advanced escalation pathway

**Table 3:** Longterm mechanical support devices. “↑” is Increases, “↓” is decreases.

Device	Mechanism of Support	Typical Flow (L/min)	Primary Hemodynamic Effects	Indications	Major Complications	Clinical Role
Continuous-Flow LVAD (HeartMate 3)	Axial/centrifugal LV-to-aorta continuous flow	4.0–6.0	↑ CO, ↓ LV filling pressures, reverse remodeling	Advanced HFrEF (BTT, DT)	Bleeding, stroke, infection, pump thrombosis (low) [59]	Gold standard durable LV support
Continuous-Flow LVAD (HVAD)	Centrifugal LV-to-aorta flow	4.0–6.0	↑ CI, ↓ PCWP	Advanced HF (historical)	Stroke, pump thrombosis [59]	Withdrawn from market; legacy patients only
BiVAD (Durable)	Separate LVAD + RVAD	4.0–6.0 (each side variable)	Biventricular unloading	End-stage BiV failure	Infection, bleeding, thrombosis [60]	Limited use; complex management
Total Artificial Heart (SynCardia)	Replacement of both ventricles	7.0–9.5	Complete biventricular replacement	Refractory biventricular failure, transplant candidates	Infection, bleeding, stroke, renal failure [60,61]	Bridge to transplant
Destination Therapy LVAD	Continuous-flow LV support	4.0–6.0	Sustained ↑ CO, ↓ congestion	Non-transplant candidates	Late bleeding, stroke, driveline infection [61]	Long-term survival therapy
Bridge-to-Transplant LVAD	Continuous-flow LV support	4.0–6.0	Hemodynamic stabilization	Transplant candidates	Bleeding, thromboembolism [61]	Bridge strategy

## Data Availability

No new data were created.

## Abbreviations

The following abbreviations are used in this manuscript:

MCS	Mechanical Support System
HF	Heart Failure
LV	Left ventricular
RV	Right Ventricular
CS	Cardiogenic Shock
BiV	Biventricular
LVEF	Left ventricular ejection fraction
NYHA	New York Heart Association
IABP	Intra-aortic pump balloon
ECMO	Extra Corporeal membrane oxygen
PCI	Percutaneous coronary intervention
LVAD	Left ventricular assist device
RVAD	Right ventricular assists device
TAH	Total artificial heart
FDA	U.S. Food and Drug Administration
CMS	Centers for Medicare and Medicaid Services
